# 2019 List of Reviewers

**DOI:** 10.1117/1.JBO.25.1.010101

**Published:** 2020-01-17

## Abstract

List of reviewers who served JBO in 2019.

The *Journal of Biomedical Optics* sincerely thanks the following individuals who served as reviewers in 2019. The success of our publication hinges on the voluntary contributions of time and energy put forth by these professionals.

Atefeh Abdolmanafi
Ibrahim Abdulhalim
Walter Akers
Prabhu Britto Albert
Sergey Alexandrov
Robert Amelard
Amirhessam Aminfar
Joseph Angelo, Jr
Md Ansari
Matthew Applegate
Vanderlei Bagnato
Wesley Baker
Oluwaseyi Balogun
Mihaela Balu
Timothy Baran
Utku Baran
Jennifer Barton
Alexey Bashkatov
Adam Bauer
Peter Baumhoff
Julien Bec
Muyinatu Bell
Paul Bernstein
Lina Bezdetnaya Bolotine
Olga Bibikova
Kostadinka Bizheva
Richard Blackmon
Ekaterina Borisova
Richard Bouchard
Nada Boustany
Audrey Bowden
Alan Boyde
Adrian Bradu
John Briers
Ralf Brinkmann
Peter Burgholzer
David Busch
Tyler Bywaters Rice
Delia Cabrera DeBuc
Fuhong Cai
Paul Campagnola
Kirby Campbell
Murat Canpolat
Xu Cao
Juliana C. Carmello
Hongwei Chen
Jyh-Cheng Chen
Mingzhou Chen
Nanguang Chen
Sungliang Chen
Ye Chen
Yu Chen
Shau Poh Chong
Ken Chu
Zhongdi Chu
Joseph Chue-Sang
Sohyun Chung
Riccardo Cicchi
Olga Conde
Lorenzo Cortese
Andrea Curatolo
Anna Cysewska-Sobusiak
Anabela Da Silva
Michael Daly
Arash Darafsheh
Hadis Dashtestani
Rupsa Datta
Scott C. Davis
Richard Day
Catharina de Lange Davies
Ana de Paula
Frederico de Sousa
Hamid Dehghani
Michele Diana
Jouke Dijkstra
Yichen Ding
Chen-Yuan Dong
Alexander Doronin
Dane Drutis
Nicholas Durr
Daniel Elson
Stanislav Emelianov
Mohsen Erfanzadeh
Thomas Ertl
Rinat Esenaliev
Karen Esmonde-White
Conor Evans
Leyuan Fang
Qianqian Fang
Qiyin Fang
Felix Fanjul-Velez
Hamid Farrokhi
Baowei Fei
Shangyuan Feng
Ting Feng
Pietro Ferraro
Reto Fiolka
Jonathan Fisher
Blaise Frederick
Ingemar Fredriksson
Paul French
Daniel Fried
Nathaniel Fried
Ling Fu
Ekaterina Galanzha
Xiaosong Gan
Grigory Gelikonov
Elina Genina
Irene Georgakoudi
Arkadiusz Gertych
Eugen Gheorghiu
Michael Giacomelli
Emily Gibson
Natalia Gladkova
Adam Glaser
Craig Goergen
Borivoj Golijanin
James Grant-Jacob
Bruno Grassi
Kate Grieve
Dirk Grosenick
Meisha Gul
Hanwen Guo
Abhijit Gurjarpadhye
Steffen Hackbarth
Nathan Hagen
Michael Hamblin
Songfeng Han
Diane Harper
Valentina Hartwig
Nathaniel Haven
Carole Hayakawa
Joseph Hayward
Wido Heeman
Christine Hendon
Matt Hepburn
Taylor Hinsdale
Kenneth Hoffmann
Martin Hofmann
Vanessa Holanda
Christopher Hoy
Chenfei Hu
Song Hu
Huangchiao Huang
Xiangrun Huang
Yong Huang
Anne Humeau-Heurtier
Jeeseong Hwang
Nicusor Iftimia
Justus Ilgner
Michael Insana
M. Shahidul Islam
Steven Jacques
Michael Jaeger
Mengyu Jia
Jingying Jiang
Shudong Jiang
Janggun Jo
Jesse V. Jokerst
Takashi Kakue
Vyacheslav Kalchenko
Mikhail Kandel
Dimitris Kapsokalyvas
Sebastian Karpf
Kavon Karrobi
Gordon Kennedy
Siddharth Khare
Nikolai Khlebtsov
Alexander Khmaladze
Aaron Kho
Alwin Kienle
Chulhong Kim
Ki Hean Kim
Myung Kim
Karsten Koenig
Kivanc Kose
Sri-Rajasekhar Kothapalli
Stefan Kroll
Yoshiaki Kubota
Manoj Kumar
Cristina Kurachi
David Kwartowitz
Sun Il Kwon
Sunkuk Kwon
Lily Lai
Horacio Lamela Rivera
Guy Lamouche
Lu Lan
Joseph Landry
Pierre Lane
Birgit Lange
Kirill Larin
Ethan LaRochelle
Jan Laufer
Du Le
Martin Leahy
Hsiang-Chieh Lee
Robert Lee
Seung Yup Lee
Terence Leung
Buhong Li
Changhui Li
Jiao Li
Lei Li
Li Li
Qingli Li
Rongguang Liang
Antonia Lichtenegger
Adam Liebert
Lothar Lilge
Norman Lippok
Liu Liu
Rui Liu
Yang Lou
Heard Lowry
Guolan Lu
Rongwen Lu
Alfredo Lucas
Andrei Lugovtsov
Jianwen Luo
Cheng Ma
Steen Madsen
Sudipta Maiti
Boris Majaron
Dolonchampa Maji
Srivalleesha Mallidi
Benjamin Maloney
Amir Manbachi
Dominik Marti
Barry Masters
Lev Matveev
David McGloin
Trevor McKee
Mike McShane
Andrei Medvedev
Mohammad Mehrmohammadi
Jerome Mertz
Rickson Mesquita
Kirk Michaelian
Daniel Milej
Takeo Minamikawa
Andreia Moco
Suman Mondal
Claudia Mota
Judith Mourant
Timothy Muldoon
Oleg Nadyarnykh
Florent Nageotte
Eman Namati
Sreyankar Nandy
Kamran Nasiriavanaki
Andrew Nattestad
John Quan Nguyen
Tan Nguyen
Addison Niemeyer
Bo Ning
Santi Nonell
Thomas O’Sullivan
Yusuke Ogura
Masato Ohmi
Jeffrey Oliver
Elizabeth Olson
Yihong Ong
Felipe Orihuela-Espina
Daniel Orringer
Cynthia Pagba
Akilan Palanisami
Nikhil Paliwal
Guenther Paltauf
Vimal Prabhu Pandiyan
Asael Papour
Seonyeong Park
YongKeun Park
Manish Patankar
Ronak Patel
Ivan Pelivanov
Zhaoqiang Peng
Rozhin Penjweini
Daqing Piao
Fabien Picot
Mark Pierce
Roberto Pilot
Michael Pircher
Andrew Plumb
Brian Pogue
Daria Pominova
Alexey Popov
Dmitry Postnov
Eric Potma
Samuel Powell
Scott Prahl
Manojit Pramanik
K Pu
Le Qiu
Kyle Quinn
Narasimhan Rajaram
Gabriel Ramirez
Julio Ramirez-San Juan
Janaka Ranasinghesagara
Fulvio Ratto
Aniruddha Ray
Karen Reiser
Liqiang Ren
Brendon Restall
Rebecca Richards-Kortum
Jose Rico-Jimenez
Jorge Ripoll
Charles Riva
Yair Rivenson
William Roach
Michael Roberts
Francisco Robles
Darren Roblyer
Jeremy Rogers
Peter Rolfe
Marek Romanowski
Joseph Rosen
Francesca Rossi Paoletti
Christopher Rowlands
Marcello Rubessa
Alberto Ruiz
Rolf Saager
Leonardo Sacconi
Negar Sadeghipour
Andrey Sagaidachyi
Sudipta Saha
Sava Sakadzic
Inga Saknite
Felix Scholkmann
Karl Schulmeister
Hinnerk Schulz-Hildebrandt
Dietrich Schweitzer
Oxana Semyachkina-Glushkovskaya
Natan Shaked
Vladislav Shcheslavskiy
Jennifer Shell
Yihui Shen
Lingyan Shi
Marina Shirmanova
Xiao Shu
Mira Sibai
Ludovico Silvestri
Kanwarpal Singh
Emil Sobol
Sergei Sokolovski
Cheol Song
Janis Spigulis
Antonello Spinelli
Bryan Q. Spring
Vivek Srinivasan
Ronald Sroka
Samuel Streeter
Iain Styles
Klaus Suhling
Chiawei Sun
Yuansheng Sun
Ulas Sunar
Nima Tabatabaei
Yidong Tan
Jianbo Tang
Yubo Tang
Chao Tao
Tanja Tarvainen

